# Comparing theory and non-theory based implementation approaches to improving referral practices in cancer genetics: a cluster randomised trial protocol

**DOI:** 10.1186/s13063-019-3457-6

**Published:** 2019-06-20

**Authors:** April Morrow, Emily Hogden, Yoon-Jung Kang, Julia Steinberg, Karen Canfell, Michael J. Solomon, James G. Kench, Anthony J. Gill, Tim Shaw, Nicholas Pachter, Bonny Parkinson, Luke Wolfenden, Gillian Mitchell, Finlay Macrae, Kathy Tucker, Natalie Taylor

**Affiliations:** 1 Cancer Council NSW, Woolloomooloo, NSW Australia; 2 University of Sydney, Sydney, NSW Australia; 3 Department of Colorectal Surgery, Royal Prince Alfred Hospital, Camperdown, NSW Australia; 4 Surgical Outcomes Research Centre (SOuRCe), Royal Prince Alfred Hospital, Camperdown, NSW Australia; 5 Department of Tissue Pathology and Diagnostic Oncology, Royal Prince Alfred Hospital, Camperdown, NSW Australia; 6 Cancer Diagnosis and Pathology Group, Kolling Institute of Medical Research, Royal North Shore Hospital St Leonards, St Leonards, NSW Australia; 7 NSW Health Pathology Department of Anatomical Pathology, Royal North Shore Hospital, St Leonards, NSW Australia; 8 Genetic Services of Western Australia, King Edward Memorial Hospital, Subiaco, WA Australia; 9 Macquarie University Centre for the Health Economy, Macquarie University, Macquarie Park, NSW Australia; 10 School of Medicine and Public Health, University of Newcastle, Callaghan, NSW Australia; 11 Familial Cancer Research Centre, Peter MacCallum Cancer Centre, Melbourne, VIC Australia; 12 Sir Peter MacCallum Department of Oncology, University of Melbourne, Melbourne, VIC Australia; 13 Colorectal Medicine and Genetics, and Department of Medicine, University of Melbourne, The Royal Melbourne Hospital, Melbourne, VIC Australia; 14 Hereditary Cancer Clinic, Prince of Wales Hospital, Syndey, NSW Australia; 15 Prince of Wales Clinical School, UNSW Sydney, Sydney, NSW Australia

**Keywords:** Lynch syndrome, Hereditary cancer, Implementation, Theoretical domains framework, Behaviour change

## Abstract

**Background:**

Lynch syndrome (LS) is an inherited, cancer predisposition syndrome associated with an increased risk of colorectal, endometrial and other cancer types. Identifying individuals with LS allows access to cancer risk management strategies proven to reduce cancer incidence and improve survival. However, LS is underdiagnosed and genetic referral rates are poor. Improving LS referral is complex, and requires multisystem behaviour change. Although barriers have been identified, evidence-based strategies to facilitate behaviour change are lacking. The aim of this study is to compare the effectiveness of a theory-based implementation approach against a non-theory based approach for improving detection of LS amongst Australian patients with colorectal cancer (CRC).

**Methods:**

A two-arm parallel cluster randomised trial design will be used to compare two identical, structured implementation approaches, distinguished only by the use of theory to identify barriers and design targeted intervention strategies, to improve LS referral practices in eight large Australian hospital networks. Each hospital network will be randomly allocated to a trial arm, with stratification by state. A trained healthcare professional will lead the following phases at each site: (1) undertake baseline clinical practice audits, (2) form multidisciplinary Implementation Teams, (3) identify target behaviours for practice change, (4) identify barriers to change, (5) generate intervention strategies, (6) support staff to implement interventions and (7) evaluate the effectiveness of the intervention using post-implementation clinical data. The theoretical and non-theoretical components of each trial arm will be distinguished in phases 4–5. Study outcomes include a LS referral process map for each hospital network, with evaluation of the proportion of patients with risk-appropriate completion of the LS referral pathway within 2 months of CRC resection pre and post implementation.

**Discussion:**

This trial will determine the more effective approach for improving the detection of LS amongst patients with CRC, whilst also advancing understanding of the impact of theory-based implementation approaches in complex health systems and the feasibility of training healthcare professionals to use them. Insights gained will guide the development of future interventions to improve LS identification on a larger scale and across different contexts, as well as efforts to address the gap between evidence and practice in the rapidly evolving field of genomic research.

**Trial registration:**

ANZCTR, ACTRN12618001072202. Registered on 27 June 2018.

**Electronic supplementary material:**

The online version of this article (10.1186/s13063-019-3457-6) contains supplementary material, which is available to authorized users.

## Background

### Evidence translation for genetics and genomics

Advances in genetic and genomic research have promised to transform future approaches to disease prevention, detection and treatment [1, 2]. In the clinical setting, the development of high-throughput technologies for genetic sequencing and analysis has generated new opportunities for improved diagnosis of genetic disorders, personalised targeted treatments (particularly for cancer patients), prenatal screening and diagnosis, pharmacogenomics and population-based assessment of disease risk [3, 4]. However, the process of integrating these changes into existing healthcare systems has been slow and challenging, with health systems struggling to keep up with the exponential speed at which the genomics evidence-base is evolving [4–6].

Next-generation sequencing platforms have improved the affordability and feasibility of detecting rare, but highly penetrant mutations in cancer predisposition genes, such as *TP53*, *BRCA1, BRCA2, APC* and the mismatch repair genes - *MLH1, MSH2, MSH6* and *PMS2* [2]. With increasing recognition of the clinical utility of genetic information, the number of patients being offered genetic testing is increasing, putting pressure on specialist genetics services that have limited capacity to cope with the increased demand [1, 6]. Some genetics-related tasks have already been deployed to clinicians (sometimes termed “mainstreaming”), for example with surgeons or oncologists now ordering genetic tests and interpreting pathology reports to determine risk and whether to refer to genetics [6–10]. This requires incorporating new practices and behaviours; however, the success of these has been mixed (e.g. with suboptimal referral rates identified) [7, 11]. With more mainstreaming of genomics into health systems on the horizon, there is a need to understand how best to incorporate genetic responsibilities into clinical practice.

### Frameworks and theory to support implementation

Implementation science uses evidence-based strategies (e.g. system change interventions, quality monitoring tools) to promote the behavioural change necessary for effective implementation, and can be applied to all translational phases of the genomic research continuum to promote uptake in the clinical setting [5, 12]. Implementation science frameworks (e.g. the consolidated framework for implementation research (CFIR) [13]; reach, effectiveness, adoption, implementation and maintenance (RE-AIM) [14]; promoting action on research implementation in health services framework (PAHRIS) [15]) consolidate these strategies to provide guided, systematic approaches that can be used by researchers and/or clinicians to enhance implementation. However, a recent literature review demonstrated that less than 2% of studies aimed at translating genetics and genomics research into clinical practice explicitly made use of these implementation science frameworks [5]. Given the pace at which genomic research is expanding, failure to utilise evidence-based implementation frameworks is likely to widen the gap between evidence and practice [5].

Implementation frameworks with a theoretical basis can enhance understanding of the factors influencing implementation success or failure and have been proven effective in producing health system behaviour change across a number of settings [16–18]. One example is the theoretical domains framework (TDF), which synthesises a range of behaviour change theories to aid the identification of behaviour change barriers, which can then be specifically targeted by evidence-based interventions [19]. The TDF has been used extensively in clinical practice to promote healthcare professional behaviour change, but with variable success [16, 20–22]. Understanding factors that mediate intervention success across complex and unpredictable health systems remains a challenge [23, 24]. The success or failure of an intervention is likely to result from a complex interplay between a number of factors, including the nature of the clinical problem; novelty of proposed changes; appropriateness of the chosen implementation framework; attributes of the individual(s) driving the change (e.g. level of experience, psychosocial determinants) and contextual influences at local, organisational and system levels [25]. In such settings, rigorous trials of different implementation approaches may help to begin unravelling these complexities.

### Implementing Lynch syndrome evidence into practice

Lynch syndrome (LS) is one of the genetic syndromes that has been impacted by these implementation challenges. LS is an autosomal dominant cancer predisposition syndrome conferring an increased risk of colorectal, endometrial and other cancer types [26]. Identifying individuals affected by LS allows access to cancer risk management strategies (e.g. colonoscopic surveillance, risk-reducing hysterectomy) proven to reduce cancer incidence and improve survival [27, 28]. Despite guidelines recommending universal LS screening strategies for patients with LS-associated cancers, the condition remains largely underdiagnosed and current genetic referral rates are suboptimal [11]. In Australia, little is known about LS diagnostic pathways or the practices hindering rates of referral - partially owing to the challenges of linking data between hospitals, pathology providers and familial cancer clinics [29], as well as a lack of standardised referral pathways upon which these data can be mapped to identify system failures. Whilst Brennan and colleagues identified various stages at which cases of colorectal cancer (CRC) slipped through the LS referral cracks in hospitals in the Australian Capital Territory [11], further work is needed to establish practices at a national level.

Recently, a pilot study [30] was conducted at two large Australian hospitals to improve the identification of LS amongst patients with CRC using the theoretical domains framework implementation (TDFI) approach: a theory-grounded, 6-step approach that combines the TDF with implementation science principles to systematically promote behaviour change in accordance with clinical guidelines [16]. The following TDFI steps were carried out at each site: (1) local multidisciplinary teams formed to map current LS referral processes; (2) target behaviours were identified using discussion supported by a retrospective audit; (3) barriers to those behaviours were identified using the validated Influences on Patient Safety Behaviours Questionnaire (IPSBQ) and TDFI-guided focus groups; (4) interventions were co-designed to address barriers using focus groups; (5) interventions were co-implemented and (6) impact of the intervention was evaluated [30]. The rationale for this approach was to actively involve healthcare professionals throughout the implementation process, particularly by using their tacit knowledge to contextualise problems and co-design pragmatic interventions [31] alongside input from implementation experts with theoretical expertise in behaviour change [32].

The pilot study yielded mixed results, with changes in some behaviours (e.g. improved ordering of supplementary pathology tests, standardised wording of pathology reports) but no overall improvement in the primary study outcome (number of LS referrals) [30]. Post-implementation interviews were conducted with healthcare professionals involved in the LS implementation pilot study, with thematic analysis highlighting key implementation challenges across both theoretical and practical boundaries. These included (1) the accessibility of theory to healthcare professionals (e.g. lack of understanding and/or perceived necessity of behaviour change theory), (2) compliance with theory by healthcare professionals (e.g. a tendency to jump straight into solution generation rather than following TDFI steps), (3) navigating the system (e.g. lengthy governance processes, lack of external researcher knowledge about internal stakeholder networks and contacts) and (4) stakeholder management (e.g. lack of researcher awareness about existing internal politics and hierarchies).

### Refining implementation approaches and testing the impact of theory

One approach to solving both the theory-related and practical problems presented is to train healthcare professionals from within the system to use an implementation science framework as part of a research study, specifically the TDFI approach in this instance. Training healthcare professionals from within the system is likely to alleviate (1) theoretical barriers (accessibility and compliance) via education about the rationale and demonstrated benefits of a theory-based approach, and upskilling to ensure the approach is applied accurately and (2) practical barriers (navigating the system and stakeholder management) by drawing on knowledge from within the system about existing processes, bureaucracy and internal stakeholder networks and politics. Not only is this likely to enhance the feasibility of the TDFI approach for improving clinical practice in genomics, it is also likely to improve the fidelity of delivery of the approach, and the rigour under which it is tested - crucial challenges that need to be addressed to advance the field of implementation science [32].

Theoretically underpinned implementation frameworks can optimise and contextualise the design of behaviour change interventions, whilst increasing opportunities to understand generalizability to other settings [33]. However, to understand the impact of the use of theory in an implementation approach, and to advance the science of behaviour change in healthcare, it is important to distinguish the theoretical components (e.g. use of the TDF to identify barriers and generate targeted intervention strategies), holding as many of the other aspects of the implementation approach constant. Therefore, this study will compare the effectiveness of two identical, structured implementation approaches delivered by trained healthcare professionals, distinguished only by the use of theory to identify barriers and to design targeted interventions, in improving clinical practice for identifying patients with LS.

## Methods

### Design

**Fig. 1:**
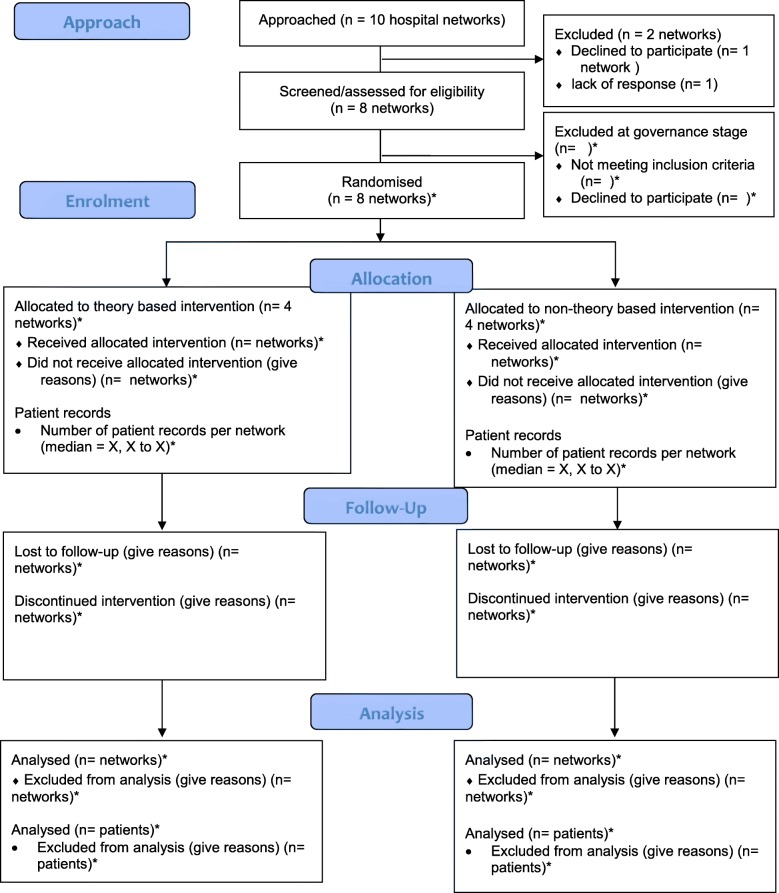
Consolidated Standards of Reporting Trials statement extension for social and psychological interventions (CONSORT-SPI) flow diagram. Diagram of participant (hospital networks) recruitment. *Numbers to be incorporated as trial progresses

This protocol is reported in line with the Standard Protocol Items: Recommendations for Interventional Trials (SPIRIT) checklist (Additional file 1) [34]. The results will be reported in line with the Consolidated Standards of Reporting Trials statement extension for social and psychological interventions (CONSORT-SPI 2018 extension) [35]. Consequently, the CONSORT-SPI flow diagram is reported in this protocol (see Fig. 1).

A parallel, cluster randomised trial will be used to test for differences between a theory-based implementation approach (TDFI) and a non-theory based implementation approach to improving LS referral practices in eight large Australian hospital networks across three states [New South Wales (n=4), Victoria (n=2), and Western Australia (n=2)]. As sharing of staff and resources in networked hospitals is likely to lead to correlations between outcomes, each hospital network will comprise a separate cluster in this trial (i.e. eight clusters in total). Hospital networks will be randomly allocated to the theory arm or non-theory arm using a computer-generated random sequence. To account for differences in region-specific LS detection pathways, the randomisation will be stratified by participating states, with the same number of networks from each state allocated to theory and non-theory arms (a 1:1 allocation ratio). A theory-based, mixed methods process evaluation will be conducted alongside the trial to investigate intervention fidelity, potential mechanisms of impact, mediators and contextual moderators, for both approaches [24, 36].

### Overview of implementation intervention

At each hospital network, a locally employed healthcare professional (see “Implementation Lead recruitment”) from within the system will be trained to lead a 7-phase implementation intervention approach (see “Procedure”).

### Outcomes

#### Pre-implementation outcomes

CRC surgery, pathology, and familial cancer clinic (FCC) clinical data will be retrospectively collected across all hospital networks for patients admitted during a 24-month period prior to study commencement, to demonstrate LS referral pathway clinical practice. The data will be cross-referenced with LS referral maps to be constructed for each hospital network. The proportion of patients who transition to each step, in adherence to the pathway map will be calculated, leading to the identification of bottlenecks. The referral maps and bottlenecks will be compared across hospital networks, with particular attention to potential differences in processes between geographical regions. A validated TDF-based questionnaire (Influences on Patient Safety Behaviours Questionnaire) [37] will be used to quantitatively evaluate psychosocial barriers to risk-appropriate completion of the LS referral pathway. Qualitative focus group data will be used to understand key barriers in context.

#### Post-implementation outcomes

The primary post-implementation quantitative outcome will be the change in the proportion of patients with risk-appropriate completion of the site-specific LS tumour testing and referral pathway within 2 months of CRC resection. Secondary outcomes will be (1) proportion of high risk-patients who were referred to the FCCs (sensitivity of referral), (2) proportion of referred patients who attended FCC and (3) proportion of patients with missing testing and referral data.

### Recruitment

#### Study site recruitment

The research team approached ten Australian hospital networks to participate. Due to the difficulty of obtaining the number of CRC resections performed by hospital per year, the eligibility criteria used to identify networks was defined as “hospitals where CRC resections are routinely performed”. A hospital network may consist of a single hospital or several hospitals (including co-located or off-site pathology laboratories and FCCs) in which staff and resources are shared. In each cluster, at least one hospital of > 500 beds is included. The Chief Investigator steering committee (see Additional file 2 for study personnel roles and responsibilities) was consulted to identify hospitals to approach to participate. Of the ten hospital networks approached, two were excluded. Research governance applications for eight networks (each consisting of 1–3 individual hospital sites) are being prepared for or reviewed by hospital research governance offices, and formal enrolment will be concluded when research governance approval is granted for each hospital network. No exact numbers of patients with colorectal cancer who undergo resection were available for the individual hospitals; rough estimates are 100–250 per hospital network per annum.

#### Implementation Lead recruitment

At each hospital network, a locally employed healthcare professional will be appointed as a paid Implementation Lead for one day per week over a 24-month period to coordinate the implementation approach and to oversee data collection. They will have a professional understanding of the LS patient pathway, and the ability to motivate other LS stakeholders to engage with implementation processes. These criteria will be used to select the most appropriate candidate for the Implementation Lead role at each hospital network.

#### LS patient pathway stakeholder recruitment

Using snowball recruitment methods, stakeholders in the LS patient referral pathway (e.g. surgeons, pathologists, oncologists, registrars, genetic counsellors) will be invited by the Implementation Lead to participate in one or more study activities relevant to their role at timepoints throughout the implementation approach. These include process map meetings, questionnaires, focus groups and intervention implementation planning meetings.

### Allocation and concealment

Block randomisation for this study was carried out at the level of the hospital networks. Each hospital network (and associated pathology laboratory and FCC) was allocated to the theory arm or non-theory arm by a member of the research team not involved in recruitment of hospital networks, using a random computer-generated sequence, with stratification by state. The research team will be aware of the trial arm allocation to train the Implementation Leads; however, the allocation will be concealed from the Implementation Leads, and the individual participants they recruit [Implementation Team members (see Phase 2) and LS patient pathway stakeholders]. Following randomised allocation, Implementation Leads were trained in one of two structured implementation approaches: either a theory-based approach or a non-theory based approach. Allocation has been concealed from the Chief Investigators. To prevent selection bias, all hospital networks were recruited prior to randomisation, and all adult patients undergoing a CRC resection at each hospital network within the study timeframe will be included in the study. Currently, there are no anticipated circumstances when unblinding would be considered permissible. Given the inability to fully control interactions between individuals at different networks involved in the trial, we accept that masking of Implementation Leads and individual participants may be compromised. Plans to investigate the success of masking amongst Implementation Leads are being incorporated into the process evaluation.

## Measures

### Clinical data collection

Patient-level clinical practice data relating to the CRC and LS referral pathway will be extracted from relevant surgical, pathology, and FCC databases by the Implementation Leads (see Additional file 2 for variables). Patient data will be collected throughout the implementation of the intervention, and outcome data will be collected for up to 9 months after the end of the intervention strategies. Patients will be eligible for inclusion if they undergo CRC resection at participating hospital networks within the time periods for data collection and are aged over 18 years at the time of resection. Given the short duration of the trial and minimal risk of harm, a formal data monitoring committee will not be required. An ethics-committee-approved data management plan has been presented to research governance officers and will be refined for each participating hospital network to ensure compliance with site-specific protocols.

### Perceived barriers

#### Questionnaire

The validated IPSBQ [37] will be used to assess barriers to the relevant target behaviour for change in relation to LS identification and referral, incorporating additional items to reflect the 14-domain TDF developed by Cane et al. (2012) [19]. Participants in the theory-based implementation arm will be provided with results from the IPSBQ to facilitate barrier identification and development of targeted intervention strategies as part of the implementation process. Participants in the non-theory trial arm will also complete the IPSBQ, but only for the purpose of measuring changes in perceived barriers pre-post intervention.

#### Theory-based focus group schedule

The content of the theory-based focus group schedules and additional materials (see Additional file 2) was developed based on the TDFI to encourage discussion of barriers explicitly in the context of the TDF [16, 38].

#### Non-theory based focus group schedule

The content of the non-theory based focus groups and additional materials (see Additional file 2) was developed to promote generic discussion of barriers based on intuition and tacit knowledge, and does not include IPSBQ results or TDF-guided questions about barriers and interventions [38].

### Procedure (detailed description of implementation intervention)

**Fig. 2:**
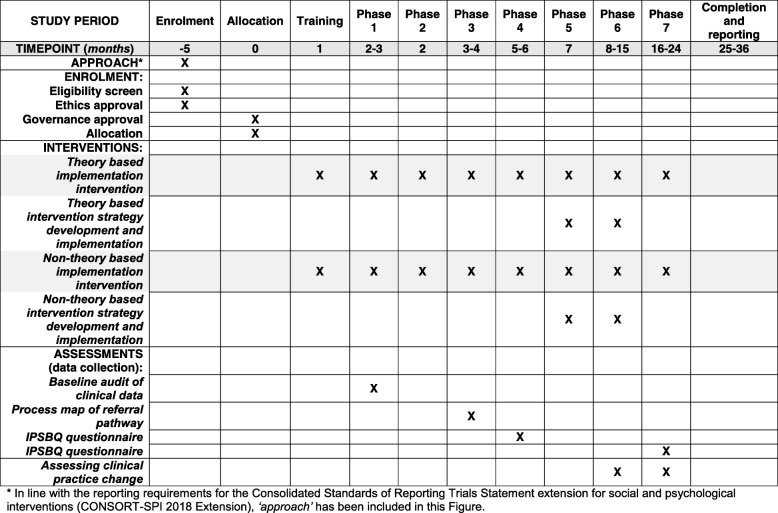
Standard Protocol Items: Recommendation for Interventional Trials (SPIRIT) diagram. Schedule for enrolment, intervention procedures and assessment. IPSBQ, Influences on Patient Safety Behaviours Questionnaire

The Implementation Lead at each site will oversee the 7 study phases described below. Phases 4 and 5 will differentiate the theory-based and non-theory based components of the implementation approaches, according to trial arm allocation; the non-theory based components have been modified from the TDFI approach [16]. Phases 1–3 and 6–7 will remain the same for both arms. The study procedures for enrolment, intervention procedures and assessment are presented in the SPIRIT diagram in Fig. 2.

#### Implementation Lead training and ongoing support

The standard training content received by both groups was developed based on common components of comprehensive implementation initiatives utilised by health services for the purposes of improvement [16, 39]. Differences in training materials used in the trial arms will be distinguished only by the inclusion of additional TDF-guided content in the theory-based implementation arm. The training will comprise an introduction to implementation science and specific instructions, practical activities and tools for moving through the 7 phases of the implementation approaches described below (see Additional file 2 for description of training contents). Implementation Leads will be informed of planned teleconference calls with the research team over the study period to support the delivery of each phase, and provided with additional tools for recording study-related activities for process evaluation purposes. These teleconferences will be used to identify and address challenges to participant recruitment and to record and manage any unintended effects of the interventions. Site-specific data management plans, including measures to protect confidentiality of individual participants, will be communicated to Implementation Leads during teleconferences.

#### Phase 1 - undertake baseline audit

The Implementation Leads at each site will undertake data extraction using the clinical practice data extraction tool to establish baseline evidence on existing LS referral practices (see Additional file 2 for data variables). These data will be de-identified and securely transported to the research team. The research team will carry out data checks and undertake site specific analyses for the purpose of presenting local results back to each site.

#### Phase 2 - form the Implementation Team

At each site, key LS patient pathway stakeholders will be identified by the Implementation Lead and invited to form an Implementation Team of 8–10 multidisciplinary change agents [16, 21, 40]. This will consist of healthcare professionals and administrators working in departments involved in the LS identification and referral pathway (including, but not limited to genetic counsellors, colorectal surgeons, pathologists, oncologists, nurses, administrative staff, multidisciplinary team (MDT) meeting coordinators). These individuals will bring expertise from various roles and departments, and therefore increase the likelihood of implementation success [41]. The Implementation Team (led by the Implementation Lead and with support from the research team) will work together through each phase and will also assist in recruiting other LS patient pathway stakeholders to relevant study activities.

#### Phase 3 - identify target behaviours for change

At each site, the Implementation Lead and Implementation Team will participate in two meetings to identify site-specific target behaviours for change. Meeting one will involve a discussion of LS referral pathways, where participants will discuss what they believe current processes and practice to be. Based on these discussions, the Implementation Lead will generate a LS referral pathway process map, which will be later refined in collaboration with the research team. These site-specific process maps will allow the research team to identify key aspects of the process to improve and any key hospital differences.

Prior to meeting two, baseline clinical data (extracted in phase 1 and analysed by the research team) will be mapped to relevant components of the referral process. During the meeting, Implementation Team members will reflect on their experiences of and beliefs about the referral process in light of the baseline clinical data, and explore possible system and/or behavioural change targets. Identifying the target for behaviour change will involve consideration of the impact of changing the behaviour on the desired outcome; whether the behaviour change is achievable; the likely impact (positive or negative) on other, related behaviours and the degree to which the behaviour can be measured [42]. The Implementation Lead and research team will use this information to confirm the target behaviour(s) for change.

#### Phase 4 - identify and confirm barriers to change

LS patient pathway stakeholders in both arms will complete the IPSBQ [37], and be invited to participate in a focus group. Focus groups will be facilitated (by the Implementation Lead) according to a theory-based or non-theory based format, depending on trial arm allocation (see Additional file 2 for focus group schedules). A member of the research team (NT) will attend meetings via video link to support discussions and elicit more in-depth responses.

##### Theory-based implementation arm

Focus group participants will be made aware that the IPSBQ assesses barriers according to theoretical domains explicitly linked to the identification of behaviours. They will be presented with the IPSBQ results from their site, discuss barriers in the context of the TDF and come to a consensus about the top 3–4 barriers overall. The research team will undertake a theory-based analysis of the focus group data to generate an initial implementation package, based on identification of intervention strategies informed by behaviour change techniques (BCTs) matched to identified barriers. This package will be discussed and refined by the Implementation Lead and the research team.

##### Non-theory based implementation arm

In contrast to the theory-based arm, participants will have completed the questionnaire for the purposes of measuring perceptions of barriers pre-post intervention, but will not be shown the results. Focus group participants will be asked to consider the confirmed target behaviour and develop intervention strategies based on intuition and tacit knowledge. Members of the research team (without expertise in coding and operationalising the TDF and BCTs but with expertise in implementation) will undertake a non-theory based analysis of the focus group data to generate an initial implementation package, based on identification of intuitive intervention strategies to address intuitively identified barriers. This package will be discussed and refined by the Implementation Lead and the research team.

#### Phase 5 - generate intervention strategies

For both arms, the Implementation Lead will invite LS patient pathway stakeholders to participate in a second focus group to identify and confirm intervention strategies. As in phase 4, focus groups will be facilitated differently according to trial arm allocation (see Additional file 2 for focus group schedules). Detailed descriptions of the intervention strategies used to address key barriers will be reported in accordance with Standards for Reporting Implementation Studies (StaRI) guidelines [43].

##### Theory-based implementation arm

Focus group participants will be presented with a table of barriers mapped to the TDF and solutions mapped to BCTs.

##### Non-theory based implementation arm

In the non-theory based arm, participants will be presented with a table of intuitively derived barriers and intuitively derived solutions.

Participants will be provided with a feasibility/impact matrix for each proposed strategy in both arms, and asked their opinions on the anticipated effort needed, feasibility and impact, guided by the APEASE criteria [38]. From this, the core intervention components will be refined.

#### Phase 6 - support staff to implement interventions

Intervention strategies generated in phase 5 will be implemented using general implementation science principles (i.e. the need for management support and commitment among target group members, use of boundary spanners, flexibility driven by local context, incorporation into established structures) [16]. With a template provided by the research team, Implementation Leads will create a report that will outline findings from phase 1–5, the intervention package and a plan for implementation. This will be submitted to senior management from departments affected by any proposed intervention strategies (e.g. CRC surgery, pathology and FCCs), and may require revision based on management feedback. Once management approves the proposed implementation plan, intervention strategies will then be presented to relevant forums (e.g. MDT meetings, pathology team meetings) to gain feedback that will allow the development of a broader structure to disseminate findings. The Implementation Lead will then work collaboratively with the Implementation Team to implement the intervention package over a 6-month period.

#### Phase 7 - evaluate intervention and assess practice and culture change

Following the 6-month intervention implementation period, LS patient pathway stakeholders from both arms will be invited to complete a second IPSBQ to assess any changes in perceived barriers to improving LS referral and diagnosis, as part of the simultaneous process evaluation. In addition, post-intervention clinical data will be extracted and compared to baseline clinical data to assess the impact of the intervention on clinical practice change, via assessment of the proportion of patients with CRC with risk-appropriate completion of the LS referral pathway.

### Analysis plan

#### Sample size and statistical power

The qualitative and descriptive outcomes (including the LS referral process maps, the descriptive baseline clinical data, the IPSBQ questionnaire results) are not amenable to sample size calculations. While it would be ideal to carry out a power calculation for the proportion of patients with risk-appropriate completion of the LS referral pathway, it is not possible in this situation due to the lack of exploratory trials in this area, including paucity of information on baseline outcomes, the number of patients per hospital and the intra-cluster coefficient (ICC) for outcomes in this trial. The power calculation will therefore be carried out once the baseline information is obtained. We also acknowledge that despite random allocation of the eight clusters, the characteristics may not be balanced between the two arms of the trial, representing a limitation of this trial.

#### Analysis of baseline clinical data

For each hospital network, the baseline clinical data extracted in phase 1 will be analysed for concordance with the hospital-specific LS referral pathway. Patients who transition through each step - in adherence to the pathway map - will be leading to the identification of bottlenecks. In the event that 100% adherence is demonstrated at baseline, investigators from the relevant site will be offered the option of continuing or withdrawing from the trial.

#### Assessing perceived barriers via questionnaire

Results from the IPSBQ will be analysed using descriptive statistics. Inter-item correlation will be tested to assess the internal consistency of each IPSBQ subscale (optimal range = 0.15–0.50). Pre-post questionnaire results from both trial arms will be used to assess changes in barriers as part of the aforementioned process evaluation.

#### Theory-based focus group data analysis to design intervention strategies

Stage 1: in line with previous approaches [16], focus group data will be thematically analysed using a deductive approach, with coding according to the TDF domains. The key barrier domains emerging from the focus groups will be cross referenced with those identified by the IPSBQ, and discrepancies noted.

Stage 2: analysis of the top 3–4 barrier domains will identify overlap with existing theories of behaviour change. These theories will be reviewed to identify experimental data that has demonstrated the mechanistic effect of specific behaviour change strategies for eliciting behaviour change. This review will be used to identify categories of BCTs that may be effective [44, 45]. These techniques will be operationalised into context-specific intervention strategies to address the key barriers to generate an initial intervention package, which will be reviewed and refined in collaboration with each Implementation Lead.

#### Non-theorybased focus group data analysis to design intervention strategies

Stage 1: inductive analysis will be applied to identify key intuitively derived barriers reported in the focus groups.

Stage 2: context-specific intervention strategies will be identified to address the key barriers to generate an initial intervention package, which will be reviewed and refined in collaboration with each Implementation Lead.

#### Focus group data analysis to refine the intervention package

Deductive analysis on the second set of focus group data will be applied in both trial arms, based on the APEASE [38] criteria, to assess the perceptions of the feasibility and impact of each proposed intervention strategy. From this, the core intervention components will be refined.

#### Assessing clinical practice change

The analysis of each dichotomous study outcome will be conducted at the individual patient level, using generalised estimating equation (GEE) adjustment for the clustering of patients within hospital networks. We will estimate the adjusted relative proportion (RR) of patients with the outcome using a generalised linear regression model with Poisson distribution, log link and robust standard errors. The exposure variable will be the implementation intervention status of the respective hospital network at the time of the patient’s CRC resection (pre or post implementation-intervention). We will carry out a two-sided test for each outcome, with the significance threshold defined using the Bonferroni correction for the number of outcomes tested. Missing testing and/or referral data will be treated as tests not carried out, or referral not completed, respectively. As covariates, we will consider time period (3-month intervals), time since implementation of intervention strategies (3-month intervals), patient age at operation, sex and cancer stage. The Quasi-Akaike information criterion will be used to select which covariates to include in the final model. To account for non-independence of within-hospital outcomes, all GEE analyses will be carried out with exchangeable working correlation structures. We will carry out sensitivity leave-one-out analyses by individually excluding hospital networks that accounted for more than 20% of the total patient numbers. If private patient status can be reliably obtained in at least six of eight trial sites, a supplementary analysis will be performed using private patient status as an additional covariate.

We note previous studies have found that a small number of clusters can lead to inflated type I error rates, and so-called small-sample corrections have been proposed to address this. However, these small-sample corrections were found to be too conservative for eight clusters [46]. Consequently, we will carry out a supplementary analysis using small-sample correction only for those outcomes that are robustly significantly associated with the implementation intervention status.

### Trial status

This study commenced in July 2017. Hospital networks have been approached to participate, have been randomised and are engaged in the governance process. Formal recruitment of hospital networks will be complete when research governance applications are approved. Recruitment of individual participants for the research activities described in phases 1–7 has not commenced, and data collection has not commenced.

## Discussion

The implementation of a systematic approach to LS identification and referral is complicated, and requires behavioural change amongst a multidisciplinary team of healthcare professionals working within a complex and unpredictable system. Results from the pilot study highlighted a number of theoretical and practical issues related to using a theory-based implementation approach to addressing the LS referral problem in this context - a possible explanation for the lack of improvement in referral overall [30]. By engaging someone from within the system to lead the implementation approach we seek to overcome these issues, enabling us to determine the extent to which the use of theory-based implementation approaches are (1) feasible in this setting and (2) more effective than approaches reliant on the intuition and tacit knowledge of healthcare professionals.

In addition to addressing a known clinical problem, this will be the first study to compare the effects of two implementation approaches, distinguished only by the use of theory to determine psychosocial barriers and intervention development to translate evidence into practice in complex healthcare systems. By analysing clinical practice data pre-post intervention, we will determine the more effective approach for increasing the proportion of patients with CRC with risk-appropriate completion of the LS referral and testing pathway, and the difference in the types of strategies generated through theory-driven and intuitive approaches taken to achieve this. This will also build on the work of Brennan and colleagues [11] to enhance understanding of LS referral and diagnostic practices in Australia.

Limitations of this study include an inability to control for differences between sites at individual, system and organisational levels, reflecting the real-world challenges of implementing practice-change interventions across diverse and unpredictable healthcare systems. These challenges emphasise the crucial need to study implementation trials to understand what works, in what contexts, why and at what costs [33]. The process evaluation, by providing valuable information for both approaches about intervention fidelity, potential mechanisms of impact, mediators and contextual moderators, will enable generalisability [24]. Time, resource and cost data will also be collected and incorporated into a microsimulation model (*Policy1*; currently validated for cervical [47] and bowel cancer [48] and undergoing development for LS^1^), as part of an economic evaluation to determine the most cost-effective approach.

Results from this study will not only guide the development of future interventions to improve LS identification on a larger scale and across different contexts, but also interventions to translate evidence-based practice changes from the rapidly evolving field of genomic research into complex, dynamic healthcare systems. Results will be shared within participating healthcare systems, and to the wider community. We will also contribute to the evolving Behaviour Change Technique Taxonomy [49], and to the behavioural change and implementation science community in advancing understanding on how to promote the effective use of implementation strategies to enhance uptake of evidence-based research.

## Additional files

Additional file 1: SPIRIT checklist. (DOC 127 kb)

Additional file 2: Study personnel roles and responsibilities; data variables; focus group schedules; overview of Implementation Lead training. (DOCX 44 kb)

Additional file 3: Master consent forms. (PDF 398 kb)

## Data Availability

The datasets (subject to ethics committee and individual hospital governance requirements), data management procedures and training materials used throughout the trial are held at the Cancer Council NSW and will be available from the corresponding author on reasonable request following trial completion.

## Abbreviations

CONSORT-SPI: Consolidated Standards of Reporting Trials statement extension for social and psychological interventions
CRC: Colorectal cancer
FCC: Familial cancer clinic
GEE: Generalised estimating equation
IPSBQ: Influences on Patient Safety Behaviours Questionnaire
LS: Lynch syndrome
MMR: Mismatch repair
MSI: Microsatellite instability
RR: Adjusted relative proportion
SPIRIT: Standard Protocol Items: Recommendation For Interventional Trials
TDF: Theoretical domains framework
TDFI: Theoretical domains framework implementation

## Footnotes

1 *Policy1*-Lynch comprises five model components, including a model of natural history of CRC in LS, model of screening for LS in incident CRC cases (i.e. identifying probands), model of screening for LS in family members (i.e. predictive genetic testing), model of prophylaxis and surveillance (prophylactic surgery, colonoscopic surveillance) and model of invasive cancer (cancer treatment and survival).
