# Supplementary material for: Comparing theory and non-theory based implementation approaches to improving referral practices in cancer genetics: a cluster randomised trial protocol
Source: Trials. 2019 Jun 20;20:373. doi: 10.1186/s13063-019-3457-6 (PMC6585019; doi:10.1186/s13063-019-3457-6)
Supplement: Supplementary file 2 — Study personnel roles and responsibilities; data variables; focus group schedules; overview of Implementation Lead training. (DOCX 44 kb) [file 13063_2019_3457_MOESM2_ESM.docx]

# Additional File 2

## Appendix 1 – Study personnel roles and responsibilities

**The research team**

The research team refers to the group of researches based at the Cancer Council NSW. Dr Natalie Taylor developed the grant application and was awarded the grants that fund the study. She is responsible for overseeing the research team who developed the study design, ethics application and study materials. April Morrow, Emily Hogden, Dr Julia Steinberg and Dr Yoon-Jung Kang contributed to the study development. Dr Natalie Taylor April Morrow, Emily Hogden, and Gabriella Tiernan undertook recruitment of hospital networks and had no involvement in allocation. Dr Julia Steinberg carried out allocation and was not involved in recruitment of hospital networks. The research team is responsible for coordinating the study and analysing data. The research team is aware of allocation.

**Chief and Associate Investigator steering committee**

The research team has sought advice from the Chief and Associate Investigator team throughout the study. The Chief Investigator team provide representation of the clinical specialties and healthcare professions who will be recruited in the study (e.g. colorectal surgery, oncology, pathology, and genetic counselling), implementation science expertise, and consumer representation via Susan Morris (Lynch Syndrome Australia). Steering committee meetings have been held with the larger team, and advice has been sought from sub-groups to inform the selection of the variables and data management plans. Ongoing contact is maintained through regular emails. Allocation is concealed to Chief Investigators.

**Site based personnel**

At each hospital network, an Implementation Lead is responsible for recruiting individual participants, convening meeting and focus groups and collecting questionnaires, and for implementing the intervention strategies (Phase 6). The Implementation Lead is responsible for extracting data in accordance with site specific data management plans and providing the research team with de-identified data sets. At each hospital network, a Principal Investigator (a department head or senior clinician) oversees the research activities conducted by the Implementation Lead. Allocation is concealed to site based personnel.

## Appendix 2 – Data Variables

| **Variables** |
| --- |
| ***Surgery (Admissions) data*** |
| Patient name |
| Patient date of birth |
| Patient MRN |
| Date of resection |
| Patient age at resection |
| Right versus Left tumor |
| Patient sex |
| Health insurance status |
| CRC stage (TNM pathological staging) |
| Was patient presented at MDT? |
| ***Pathology data*** |
| Date stamp of specimen receipt at pathology |
| Number of specimens received |
| Was IHC performed? |
| Results of IHC |
| Date of IHC report |
| Was MSI testing performed |
| Results of MSI testing |
| Date of MSI report |
| Was BRAF V600E testing performed? (Usually carried out if IHC staining was abnormal) |
| Results of BRAF V600E testing? |
| Date of BRAF V600E testing report |
| Was BRAF V600E performed as a reflex or supplementary test? |
| Who requested BRAF V600E testing (if supplementary) |
| Was MLH1 promoter methylation carried out? (Usually carried out if IHC staining was abnormal) |
| Results of MLH1 promoter methylation |
| Were any additional supplementary results included? |
| If so, what are they? |
| Comments made by reporting pathologist about MMR status? |
| ***Accessing pathology report and referrals*** |
| Which clinician accessed the report |
| Was the patient referred to an FCC? |
| If so, what was the date of the FCC referral? |
| Who was the referral made by? |
| Did the patient accept/decline the FCC referral? |
| ***Family Cancer Clinic (FCC) data*** |
| Date of referral to FCC |
| Who was the referral made by? |
| Did the patient attend the FCC? |
| When did they attend for the first time? |
| Were DNA germline tests ordered for the patient? |
| Were DNA germline tests conducted for the patient? |
| Date germline test ordered? |
| Date stamp of Germline tests? (e.g. report date) |
| Which genes were tested for germline DNA variants? |
| Were any pathogenic or likely pathogenic variants reported? |
| Were any variants of unknown significance reported? |
| Number of relatives who underwent predictive testing |

## Appendix 3 – Focus Group Schedules

**NB: The following additional materials will be provided to participants to facilitate these discussions: Influences on Patient Safety Behaviours Questionnaire (IPSBQ) barriers mapped to TDF domains (focus group 1), table of barriers mapped to the TDF and solutions mapped to evidence-based behaviour change techniques (focus group 2), and a feasibility/impact matrix to assess participants’ perceptions of intervention strategies in accordance with the APEASE criteria (Affordability, Practicality, Effectiveness and cost-effectiveness, Acceptability, Side effects and safety, and Equity) (focus group 2) (38).*

*The following additional materials will be used to facilitate non theory group discussions: a table of intuitively derived barriers and intuitively derived solutions (focus group 2), and a feasibility/impact matrix to assess participants’ perceptions of intervention strategies in accordance with the APEASE criteria (focus group 2)*

### Focus Group 1 (Identifying barriers)

1. Introduction, background & context

- Introduce topic: practitioner barriers to (*Insert target behaviour*)
- State purpose of individual interview/focus groups (e.g. for intervention development)
- Recording of discussion, voluntary participation and right to withdraw
- Participants to briefly introduce themselves and their role
- Describe process of doing the target behaviour and the problem to be solved

****Theory group**:**

- Describe TDF domains and how they relate to barriers; remind them about the IPSBQ which they may have completed and encourage a discussion about which barriers they think were the strongest based on IPSBQ results and why. Present the top barriers according to the IPSBQ and discuss similarities and differences in the barriers identified and the IPSBQ results, achieve consensus on top 3-4 barriers.

**Non-theory group:**

- Explain that we want to know about barriers to doing (*Insert target behaviour*).

2. Define problem:

- Define problem behaviour: (*Insert target behaviour*)

****Theory group**:**

- Explain common approaches to implementing evidence into practice, the behaviour change gap and the TDFI approach to address the LS referral problem

**Non-theory group:**

- Explain the approach the team is using to attempt to address the LS referral problem

3. Barriers to performing behaviour

****Theory group**:**

1. Present TDF barrier definitions:
   1. Encourage discussion about each type of barrier in the context of (*Insert target behaviour*)
   2. Ask participants to rate the top 4-5 they believe exist within their context and why
2. Present IPSBQ results:
   1. Determinant scores were ranked (based on number of times rated as a strong barrier by individuals) to assess different aspects of implementation difficulties. Mean scores for each determinant were also computed
   2. Discuss similarities/differences between barriers they identified and IPSBQ results
3. Arrive at a general consensus and create definitive list of top 3-4 barriers to target

| Top ranked barriers pre-IPSBQ | IPSBQ top barriers | Final list of top 3-4 barriers |
| --- | --- | --- |
|  |  |  |
|  |  |  |
|  |  |  |
|  |  |  |

**Non-theory group:**

1. Encourage general discussion about barriers to doing (*Insert target behaviour*)
   1. Ask participants to rate the top 3-4 they believe exist within their context and why
2. Arrive at a general consensus and decide which areas they think we should target
   1. Create definitive list of top 3-4 barriers using the table

|  | Final list of top 3-4 barriers |
| --- | --- |
| 1 |  |
| 2 |  |
| 3 |  |
| 4 |  |
|  |  |

### Focus Group 2 (reviewing interventions)

1. Introduction, background & context

- Introduce topic: interventions aimed at addressing barriers to (*Insert target behaviour*)
- Remind participants of top 3-4 barriers to (*Insert target behaviour*)
- State purpose of individual interview/focus groups (e.g. to review intervention package, etc.)
- Recording of discussion, voluntary participation and right to withdraw
- Participants to briefly introduce themselves and their role

2. Focus group procedure

****Theory group**:**

- Inform participants what we did with the information gathered at the first meeting:
  1. Reviewed suggestions for interventions to address identified barriers to target behaviour

|  | Top barrier in context | TDF domain | Behaviour change strategy represented | Suggested intervention strategies | Likely impact of strategy (high/moderate/ low)* | Likely feasibility of strategy (difficult/ possible)* | Ranking of intervention strategy (1 being most favourable) |
| --- | --- | --- | --- | --- | --- | --- | --- |
| Referring CRC patients at high risk of Lynch Syndrome into genetics services | E.g., Forget to remind myself to refer patient when they are well enough | Memory, attention and decision making processes | E.g., Teach to use prompts/ cues^#^ | E.g. Generate EMR alert that will appear on-screen before appointment with reminder to refer patient to FCC |  |  |  |
|  |  |  | E.g., Use of follow up prompts^ | E.g. Genetic counsellor to send monthly email to surgeons & oncologists with list of high-risk LS patients for whom referral not yet received |  |  |  |
|  |  |  |  | Ideas from focus group 1 data synthesis |  |  |  |
|  |  |  |  | Ideas from focus group 1 data synthesis |  |  |  |
|  |  |  |  | Space for more ideas |  |  |  |

- 1. Mapped against theory
  2. Used theory to guide additional intervention development
  3. Created a matrix with impact/feasibility columns included.
- Provide a copy of matrix and ask participants to review the interventions and likely impact/feasibility
- Refine core intervention components against the identified barriers

*Refer to APEASE criteria;

#Teach the person to identify environmental prompts which can be used to remind them to perform the behaviour. This could include times of day, particular contexts or elements of contexts which prompt them to perform the target behaviour;

^Involves sending letters, making telephone calls, visits or follow up meetings after the major part to the behaviour change intervention has been completed.

**Non-theory group:**

Inform participants what we did with the information gathered at the first meeting:

a. Reviewed suggestions for interventions to address identified barriers to target behaviour

- 1. Developed interventions
  2. Created a matrix with impact/feasibility columns included.

Provide a copy of matrix and ask participants to review the interventions and likely impact/feasibility

Refine core intervention components against the identified barriers

|  | Top barrier in context | Suggested intervention strategies | Likely impact of strategy (high/moderate/ low)* | Likely feasibility of strategy (difficult/ possible)* | Ranking of intervention strategy (1 being most favourable) |
| --- | --- | --- | --- | --- | --- |
| Referring CRC patients at high risk of Lynch Syndrome into genetics services | E.g., Forget to remind myself to refer patient when they are well enough | E.g. Put a note in patient file as reminder for next appointment |  |  |  |
|  |  | E.g. ask nurse coordinator to remind me to refer patient when well enough |  |  |  |
|  |  | Ideas from focus group 1 data synthesis |  |  |  |
|  |  | Ideas from focus group 1 data synthesis |  |  |  |
|  |  | Space for more ideas |  |  |  |
|  |  | Space for more ideas |  |  |  |

*Refer to APEASE criteria

## Appendix 4 – Overview of Implementation Lead training contents

The training materials for the following content were adapted from the ‘ABC for Patient Safety’ Toolkit (<http://www.improvementacademy.org/resources/abc-for-patient-safety-workshop-and-toolkit/>) developed by CIA Taylor and colleagues from the Bradford Institute for Health Research.

*NB: Session objectives are presented, and an indication of the implementation approach ‘phases’ are provided. An outline of the additional information provided for the theory based group is highlighted throughout.*

Session 1: Background and rationale

- Provide an overview of Lynch syndrome
- Discuss the Hide and Seek Project aims
- Present barriers to practice change in health systems and Lynch syndrome referral
- Introduce Implementation Science as a way to address barriers
- Activity: discuss barriers to implementing evidence into clinical practice

****Theory group only**:**

- Provided with information about behaviour change theory and how it can help to predict behaviour and inform the design of interventions to elicit behaviour change in different settings

Session 2: Changing clinical practice behaviours: the case of nasogastric tubes

- Discuss why changing clinical practice is difficult
- Present an example of successful practice change (nasogastric tubes) using an implementation science approach
- Demonstrate the evidence and benefits of an implementation science intervention

****Theory group only**:**

- Provided information about how the Theoretical Domains Framework (TDF) was used to address a nasogastric tube patient safety issue, and development and history of the Theoretical Domains Framework Implementation approach

Session 3: Overview of Implementation Lead role (Phase 2)

- Provide an overview of the Implementation Lead role
- Discuss ways to promote collaborative learning and inclusivity
- Work through Phase 2: Form Implementation Teams
- Sketch plans for taking Phase 2 forward at your site

Session 4: Identifying target behaviours (Phase 3)

- Understand the importance of identifying a target behaviour
- Identify the difference between behaviours and goals
- Develop skills for identifying target behaviours

Session 5: Identifying barriers to practice change (Phase 4) and generating solutions (Phase 5)

- Understand the importance of identifying barriers for designing interventions
- Develop skills for running focus groups to identify barriers and develop intervention strategies
- Sketch plans for taking Phase 4 and 5 forward at your site

****Theory group only**:**

- Use of Influences on Patient Safety Behaviours Questionnaire (IPSBQ) to assess barriers (e.g., knowledge, environment/resources, memory, and emotion) to performing the target behaviour(s) according to the TDF domains,
- Running focus groups using a TDF based interview schedule
- Introduction to the process of mapping barriers to the TDF and use of behaviour change techniques to generate tailored solutions

Session 6: Implementing intervention strategies (Phase 6)

- Understand the importance of systematically planning intervention implementation with stakeholders
- Develop knowledge about how to plan intervention implementation
- Describe the process for taking Phase 6 forward

Session 7: Data extraction training (Phases 1 and 7)

- Understand the importance of systematically planning intervention implementation with stakeholders
- Develop knowledge about how to plan intervention implementation
- Describe the process for taking Phase 6 forward

Session 8: Process evaluation

- Rationale for process evaluation alongside trial
- Overview of process evaluation measures (e.g. stakeholder interviews, observation of meetings and focus-groups, project log)
- Project log and handover processes

****Theory group only**:**

- Rationale for theory-based process evaluation, process evaluation data to be mapped to TDF to identify theoretical determinants of behaviour change

Session 9: Conclusion

- Overview of ongoing structured support provided by Cancer Council NSW research team
- Discussion and workshopping of any anticipated challenges
