# Supplementary material for: Comparing theory and non-theory based implementation approaches to improving referral practices in cancer genetics: a cluster randomised trial protocol
Source: Trials. 2019 Jun 20;20:373. doi: 10.1186/s13063-019-3457-6 (PMC6585019; doi:10.1186/s13063-019-3457-6)
Supplement: Supplementary file 3 — Master consent forms. (PDF 398 kb) [file 13063_2019_3457_MOESM3_ESM.pdf]

PARTICIPANT INFORMATION SHEET: IMPLEMENTATION TEAMS

Hide and Seek with hereditary cancer: Improving detection of colorectal cancer patients with a high risk of Lynch Syndrome

Invitation
You are invited to participate in a research study seeking to improve processes around identifying cancer patients with Lynch Syndrome through timely referral to genetic testing.

The study is being conducted by:
- Dr Natalie Taylor, Cancer Council NSW
- Ms April Morrow, Cancer Council NSW
- Dr Yoon-Jung Kang, Cancer Council NSW
- Dr Julia Steinberg, Cancer Council NSW
- [insert site specific Principal Investigator name and hospital]

Before you decide whether or not you wish to participate in this study, it is important for you to understand why the research is being done and what it will involve. Please take the time to read the following information carefully and discuss it with others if you wish.

1. What is the purpose of this study?
Lynch syndrome (LS) is a cancer predisposition syndrome (caused by an inherited mutation in a mismatch repair gene) that confers an increased risk of colorectal, endometrial and other cancer types. Approximately 2-5% of colorectal cancer (CRC) patients have an underlying mutation causing LS. The condition is believed to affect tens-of-thousands of Australians, but is extremely underdiagnosed. Early diagnosis of LS is critical since cancer risk management strategies (e.g., colonoscopic surveillance, hysterectomy and salpingo-oophorectomy) for LS patients and at-risk relatives can prevent cancer diagnoses and improve survival.

Whilst clinicians cannot be expected to have detailed knowledge about LS, current Australian guidelines emphasise their responsibility for recognising the clinical phenotype and family history characteristics of LS, and referring patients to clinical genetics or familial cancer services if deemed necessary. However, over the past decade, local and international studies have reported that only a small proportion of individuals with suspected LS were referred for genetic consultation and possible genetic testing.

Cancer Council NSW

This study aims to increase the number of colorectal cancer patients with a high risk of Lynch Syndrome who are referred for genetic testing at your hospital or health service. The study will also assess the implementation approach used at your hospital in a number of ways (more information on page 4). This study is planned to be conducted at eight hospitals in three states.

2. **Why have I been invited to participate in this study?**
You are eligible to participate in this study because you provide a health service to patients with colorectal cancer or have some expertise or experience around current LS clinical processes or practices.

3. **What does participation in this study involve?**
If you agree to participate in this study you will be asked to be part of an Implementation Team based at your hospital. This will be a multidisciplinary team to provide expertise from a number of viewpoints. Each Implementation Team will have an Implementation Lead (contact details of whom are available at the end of this information sheet). There are seven phases in this 2-year study. *Phases 1 and 2* will be complete when the Implementation Team is recruited. The Implementation Team will be involved at different time points in the following phases:

- *Phase 3*: Implementation Team members will participate in two one-hour meetings to 1) map current processes around identification of patients with LS and 2) use de-identified colorectal cancer patient data to identify gaps in these processes. These meetings will be audio recorded and the Implementation Lead will take notes. Everything said in a meeting is confidential so no one will be named in wider reporting of results.
- *Phase 4-5*: Implementation Team members will work within their organisation (with support from the Implementation Lead) to:
  - facilitate dissemination amongst relevant colleagues of a 5-minute questionnaire about the LS patient pathway
  - encourage colleagues to participate in two one-hour focus groups about LS.
- *Phase 6*: Implementation Team members will support the Implementation Lead to implement strategies to improve referral and testing of suspected LS carriers (‘intervention package’). Implementation Team members will participate in a one-hour meeting to plan how to provide support for the intervention. These meetings will be audio recorded and the Implementation Lead will take notes.
- *Phase 7*: After the intervention is delivered, Implementation Team members will help to disseminate a second 5-minute questionnaire to assess change in practice

The Implementation Lead will advise you of the timeline for these phases at your hospital. They will also provide you with more information about the study phases.

The Implementation Lead may invite Implementation Team members to complete the 5-minute questionnaire and/or participate in the focus groups mentioned above. If the Implementation Lead invites you to participate, they will provide you with additional information sheets and consent forms for the questionnaire and/or focus groups.

4. What if I don't want to take part in this study, or if I want to withdraw later?
Participation in this study is voluntary. It is completely up to you whether or not you participate. If you wish to withdraw from the study once it has started, you can do so at any time without having to give a reason.

5. How is this study being paid for?
The study has been jointly funded by Cancer Australia and Cancer Institute NSW.

6. Are there risks to me in taking part in this study?
No. As we are exploring current practice and clinical processes it is unlikely to be any risks. Information obtained from participants will be confidential and de-identified.

7. Will I benefit from the study?
There may be opportunities for you to earn CPD points by participating in the research or if you are considering research of your own we are open to discussing opportunities to collaborate. Certificates will be provided to all Implementation Team members and focus group participants.

In addition, you will be working to improve the referral rate for genetic testing of CRC patients with a high-likelihood risk of LS. For those CRC patients who carry LS mutations, earlier detection increases the opportunity and frequency for targeted surveillance for various relevant potential cancers which might otherwise go undetected until they are late stage.

Another benefit from this study will be the possibility of networking with other clinical professionals across the organisation and particularly between genetics and other clinical areas.

8. Will taking part in this study cost me anything, and will I be paid?
Participation in this study will not cost you anything, nor will you be paid.

9. How will my confidentiality be protected?
Any identifiable information that is collected about you in connection with this study such as comments made in meetings will be de-identified in any reporting.

10. What happens with the results?

De-identified patient data, notes and audio recordings from study meetings and focus groups, and questionnaire data will be used to in the study to:

1) Assess change in practice at your hospital
2) Co-develop an intervention package for your hospital
3) Assess the implementation approach used at your hospital to learn more about how change occurs
4) Assess the implementation approach used at your hospital and understand if it is cost-effective

Results from your hospital will be used to develop materials for study meetings and focus groups, and will be used to develop reports for colleagues and management. Results from the whole project will be published in peer-reviewed journals and presented at national and international conferences. Any information that could be used to identify individual patients or staff members will be removed by before it is used in study materials, reports, publications and presentations.

11. What should I do if I want to discuss this study further before I decide?

When you have read this information, your Implementation Lead will discuss it with you and any queries you may have. If you would like to know more at any stage, please do not hesitate to contact [insert details here] or [insert details here]

12. Who should I contact if I have concerns about the conduct of this study?

This study has been approved by the [insert HREC name here]. Any person with concerns or complaints about the conduct of this study should contact the Executive Officer on [insert details here] and quote protocol number [insert details here].

The conduct of this study at the [name of hospital] has been authorised by the [name of Local Health District]. Any person with concerns or complaints about the conduct of this study may also contact the Research Governance Officer [or other officer] on [telephone number] and quote protocol number [insert local protocol number].

Thank you for taking the time to consider this study.
If you wish to take part in it, please sign the consent form.
This information sheet is for you to keep.

CONSENT FORM

To be used in conjunction with a participant information sheet

Hide and Seek with hereditary cancer: Improving detection of colorectal cancer patients with a high risk of Lynch Syndrome

1. I,..of.. .... agree to participate in the study described in the participant information statement about joining an implementation team a part of this study.
2. I acknowledge that I have read the participant information statement, which explains why I have been selected, the aims of the study and the nature and the possible risks of the investigation, and the statement has been explained to me to my satisfaction.
3. Before signing this consent form, I have been given the opportunity of asking any questions relating to any possible physical and mental harm I might suffer as a result of my participation and I have received satisfactory answers.
4. I understand that I can withdraw from the study at any time without prejudice to my relationship with ……………………………………... Hospital, and/or my employer (potential and otherwise).
5. I agree that research data gathered from the results of the study may be published, provided that I cannot be named.
6. I understand that if I have any questions relating to my participation in this research, I may contact the research team at Cancer Council NSW on [insert details here], who will be happy to answer them.
7. I acknowledge receipt of a copy of this consent form and the participant information statement.

Complaints may be directed to [insert details here]

**Signature of participant**	**Please PRINT name**	**Date**
______________________	______________________	________________
**Signature of witness**	**Please PRINT name**	**Date**
______________________	______________________	________________
**Signature of Research Coordinator**	**Please PRINT name**	**Date**
______________________	______________________	________________

Cancer Council NSW

PARTICIPANT INFORMATION SHEET: QUESTIONNAIRE

Hide and Seek with hereditary cancer: Improving detection of colorectal cancer patients with a high risk of Lynch Syndrome

Invitation
You are invited to participate in a research study seeking to improve processes around identifying cancer patients with Lynch Syndrome through timely referral to genetic testing.

The study is being conducted by:
- Dr Natalie Taylor, Cancer Council NSW
- Ms April Morrow, Cancer Council NSW
- Dr Yoon-Jung Kang, Cancer Council NSW
- Dr Julia Steinberg, Cancer Council NSW
- [insert site specific Principal Investigator name and hospital]

Before you decide whether or not you wish to participate in this study, it is important for you to understand why the research is being done and what it will involve. Please take the time to read the following information carefully and discuss it with others if you wish.

1. What is the purpose of this study?

Lynch syndrome (LS) is a cancer predisposition syndrome (caused by an inherited mutation in a mismatch repair gene) that confers an increased risk of colorectal, endometrial and other cancer types. Approximately 2-5% of colorectal cancer (CRC) patients have an underlying mutation causing LS. The condition is believed to affect tens-of-thousands of Australians, but is extremely underdiagnosed. Early diagnosis of LS is critical since cancer risk management strategies (e.g., colonoscopic surveillance, hysterectomy and salpingo-oophorectomy) for LS patients and at-risk relatives can prevent cancer diagnoses and improve survival.

Whilst clinicians cannot be expected to have detailed knowledge about LS, current Australian guidelines emphasise their responsibility for recognising the clinical phenotype and family history characteristics of LS, and referring patients to clinical genetics or familial cancer services if deemed necessary. However, over the past decade, local and international studies have reported that only a small proportion of individuals with suspected LS were referred for genetic consultation and possible genetic testing.

This study aims to increase the number of colorectal cancer patients with a high risk of Lynch Syndrome who are referred for genetic testing at your hospital or health service. The study will also assess the implementation approach used at

your hospital in a number of ways (more information on page 4). This study is planned to be conducted at eight hospitals in three states.

2. **Why have I been invited to participate in this study?**
   You are eligible to participate in this study because you provide a health service to patients with colorectal cancer or have some expertise or experience around current LS clinical processes or practices.

3. **What does participation in this study involve?**
   If you agree to participate in this study you will be asked to complete a five-minute questionnaire (called the *Influences on Patient Safety Behaviours Questionnaire*) that will be distributed to relevant healthcare professionals before and after implementation to identify current barriers to referral, and change in the perceptions of barriers to referral.

4. **What if I don't want to take part in this study, or if I want to withdraw later?**
   Participation in this study is voluntary. It is completely up to you whether or not you participate. If you wish to withdraw from the study once it has started, you can do so at any time without having to give a reason.

5. **How is this study being paid for?**
   The study has been jointly funded by Cancer Australia and Cancer Institute NSW.

6. **Are there risks to me in taking part in this study?**
   No. As we are exploring current practice and clinical processes it is unlikely to be any risks. Information obtained from participants will be confidential and de-identified.

7. **Will I benefit from the study?**
   You will be working to improve the referral rate for genetic testing of colorectal cancer patients with a high-likelihood risk of LS. For those CRC patients who carry LS genes, earlier detection increases the opportunity and frequency for targeted surveillance for various relevant potential cancers which might otherwise go undetected until they are late stage.

   Another benefit from this study will be the possibility of networking with other clinical professionals across the organisation and particularly between genetics and other clinical areas.

8. **Will taking part in this study cost me anything, and will I be paid?**
   Participation in this study will not cost you anything, nor will you be paid.

9. **How will my confidentiality be protected?**
   We will not be collecting identifiable information as part of this questionnaire.

10. **What happens with the results?**

Cancer Council NSW

De-identified patient data, notes and audio recordings from study meetings and focus groups, and questionnaire data will be used to in the study to:

1) Assess change in practice at your hospital
2) Co-develop an intervention package for your hospital
3) Assess the implementation approach used at your hospital to learn more about how change occurs
4) Assess the implementation approach used at your hospital and understand if it is cost-effective

Results from your hospital will be used to develop materials for study meetings and focus groups, and will be used to develop reports for colleagues and management. Results from the whole project will be published in peer-reviewed journals and presented at national and international conferences. Any information that could be used to identify individual patients or staff members will be removed by before it is used in study materials, reports, publications and presentations.

11. What should I do if I want to discuss this study further before I decide?

When you have read this information, your Implementation Lead will discuss it with you and any queries you may have. If you would like to know more at any stage, please do not hesitate to contact [insert details here] or [insert details here]

12. Who should I contact if I have concerns about the conduct of this study?

This study has been approved by the [insert HREC name here]. Any person with concerns or complaints about the conduct of this study should contact the Executive Officer on [insert details here] and quote protocol number [insert details here].

The conduct of this study at the [name of hospital] has been authorised by the [name of Local Health District]. Any person with concerns or complaints about the conduct of this study may also contact the Research Governance Officer [or other officer] on [telephone number] and quote protocol number [insert local protocol number].

Thank you for taking the time to consider this study.
If you wish to take part in it, please sign the consent form.
This information sheet is for you to keep.

CONSENT FORM

To be used in conjunction with a participant information sheet

Hide and Seek with hereditary cancer: Improving detection of colorectal cancer patients with a high risk of Lynch Syndrome

1. I,..of..
.... agree to participate in the study described in the participant information statement about completing a pen and paper questionnaire.
2. I acknowledge that I have read the participant information statement, which explains why I have been selected, the aims of the study and the nature and the possible risks of the investigation, and the statement has been explained to me to my satisfaction.
3. Before signing this consent form, I have been given the opportunity of asking any questions relating to any possible physical and mental harm I might suffer as a result of my participation and I have received satisfactory answers.
4. I understand that I can withdraw from the study at any time without prejudice to my relationship with …………………………………... Hospital, and/or my employer (potential and otherwise).
5. I agree that research data gathered from the results of the study may be published, provided that I cannot be named.
6. I understand that if I have any questions relating to my participation in this research, I may contact the research team at Cancer Council NSW [insert details here], who will be happy to answer them.
7. I acknowledge receipt of a copy of this consent form and the participant information statement.

Complaints may be directed to [insert details here]

**Signature of participant**	**Please PRINT name**	**Date**
______________________	______________________	________________
**Signature of witness**	**Please PRINT name**	**Date**
______________________	______________________	________________
**Signature of Research Coordinator**	**Please PRINT name**	**Date**
______________________	______________________	________________

Cancer Council NSW

PARTICIPANT INFORMATION SHEET: FOCUS-GROUP PARTICIPATION

Hide and Seek with hereditary cancer: Improving detection of colorectal cancer patients with a high risk of Lynch Syndrome

Invitation
You are invited to participate in a research study seeking to improve processes around identifying cancer patients with Lynch Syndrome through timely referral to genetic testing.

The study is being conducted by:
- Dr Natalie Taylor, Cancer Council NSW
- Ms April Morrow, Cancer Council NSW
- Dr Yoon-Jung Kang, Cancer Council NSW
- Dr Julia Steinberg, Cancer Council NSW
- [insert site specific Principal Investigator name and hospital]

Before you decide whether or not you wish to participate in this study, it is important for you to understand why the research is being done and what it will involve. Please take the time to read the following information carefully and discuss it with others if you wish.

1. What is the purpose of this study?
Lynch syndrome (LS) is a cancer predisposition syndrome (caused by an inherited mutation in a mismatch repair gene) that confers an increased risk of colorectal, endometrial and other cancer types. Approximately 2-5% of colorectal cancer (CRC) patients have an underlying mutation causing LS. The condition is believed to affect tens-of-thousands of Australians, but is extremely underdiagnosed. Early diagnosis of LS is critical since cancer risk management strategies (e.g., colonoscopic surveillance, hysterectomy and salpingo-oophorectomy) for LS patients and at-risk relatives can prevent cancer diagnoses and improve survival.

Whilst clinicians cannot be expected to have detailed knowledge about LS, current Australian guidelines emphasise their responsibility for recognising the clinical phenotype and family history characteristics of LS, and referring patients to clinical genetics or familial cancer services if deemed necessary. However, over the past decade, local and international studies have reported that only a small proportion of individuals with suspected LS were referred for genetic consultation and possible genetic testing.

This study aims to increase the number of colorectal cancer patients with a high risk of Lynch Syndrome who are referred for genetic testing at your hospital or

Cancer Council NSW

health service. The study will also assess the implementation approach used at your hospital in a number of ways (more information on page 3). This study is planned to be conducted at eight hospitals in three states.

2. Why have I been invited to participate in this study?
You are eligible to participate in this study because you provide a health service to patients with colorectal cancer or have some expertise or experience around current LS clinical processes or practices.

3. What does participation in this study involve?
If you decide to participate, you will be asked to join in one or two one-hour focus groups. The focus groups will be run by the Implementation Lead (contact details of whom are available at the end of this information sheet), a hospital staff member who is coordinating this study at your hospital.

In the first focus group, the Implementation Lead will ask participants to discuss opinions about the key barriers to clinical practice change to improve the referral of Lynch syndrome carriers at your hospital. In the second focus group, the Implementation Lead will ask participants to review suggested solutions to barriers (interventions).

These focus groups will be audio recorded and the Implementation Lead will take notes. Everything said in a meeting is confidential so no one will be named in wider reporting of results.

4. What if I don't want to take part in this study, or if I want to withdraw later?
Participation in this study is voluntary. It is completely up to you whether or not you participate. If you wish to withdraw from the study once it has started, you can do so at any time without having to give a reason.

5. How is this study being paid for?
The study has been jointly funded by Cancer Australia and Cancer Institute NSW.

6. Are there risks to me in taking part in this study?
No. As we are exploring current practice and clinical processes it is unlikely to be any risks. Information obtained from participants will be confidential and de-identified.

7. Will I benefit from the study?
While there may not be any direct benefits from participating in the study, you will be working to improve the referral rate for genetic testing of CRC patients with a high-likelihood risk of LS. For those CRC patients who carry LS genes, earlier detection increases the opportunity and frequency for targeted surveillance for various relevant potential cancers which might otherwise go undetected until they are late stage.

Cancer Council NSW

Another benefit from this study will be the possibility of networking with other clinical professionals across the organisation and particularly between genetics and other clinical areas.

8. Will taking part in this study cost me anything, and will I be paid?
Participation in this study will not cost you anything, nor will you be paid.

9. How will my confidentiality be protected?
Any identifiable information that is collected about you in connection with this study such as comments made in a focus group will be de-identified in any reporting.

10. What happens with the results?
De-identified patient data, notes and audio recordings from study meetings and focus groups, and questionnaire data will be used to in the study to:

1) Assess change in practice at your hospital
2) Co-develop an intervention package for your hospital
3) Assess the implementation approach used at your hospital to learn more about how change occurs
4) Assess the implementation approach used at your hospital and understand if it is cost-effective

Results from your hospital will be used to develop materials for study meetings and focus groups, and will be used to develop reports for colleagues and management. Results from the whole project will be published in peer-reviewed journals and presented at national and international conferences. Any information that could be used to identify individual patients or staff members will be removed by before it is used in study materials, reports, publications and presentations.

11. What should I do if I want to discuss this study further before I decide?
When you have read this information, your Implementation Lead will discuss it with you and any queries you may have. If you would like to know more at any stage, please do not hesitate to contact [insert details here] or [insert details here]

12. Who should I contact if I have concerns about the conduct of this study?
This study has been approved by the [insert HREC name here]. Any person with concerns or complaints about the conduct of this study should contact the Executive Officer on [insert details here] and quote protocol number [insert details here].

The conduct of this study at the [name of hospital] has been authorised by the [name of Local Health District]. Any person with concerns or complaints about the conduct of this study may also contact the Research Governance Officer [or other officer] on [telephone number] and quote protocol number [insert local protocol number].

Thank you for taking the time to consider this study.
If you wish to take part in it, please sign the consent form.
This information sheet is for you to keep.

CONSENT FORM

To be used in conjunction with a participant information sheet

Hide and Seek with hereditary cancer: Improving detection of colorectal cancer patients with a high risk of Lynch Syndrome

1. I,..of... .... agree to participate in the study described in the participant information statement about being part of a focus group based at your hospital which will be audio recorded.
2. I acknowledge that I have read the participant information statement, which explains why I have been selected, the aims of the study and the nature and the possible risks of the investigation, and the statement has been explained to me to my satisfaction.
3. Before signing this consent form, I have been given the opportunity of asking any questions relating to any possible physical and mental harm I might suffer as a result of my participation and I have received satisfactory answers.
4. I understand that I can withdraw from the study at any time without prejudice to my relationship with …………………………………….... Hospital, and/or my employer (potential and otherwise).
5. I agree that research data gathered from the results of the study may be published, provided that I cannot be named.
6. I understand that if I have any questions relating to my participation in this research, I may contact the research team at Cancer Council NSW on [insert details here], who will be happy to answer them.
7. I acknowledge receipt of a copy of this consent form and the participant information statement.

Complaints may be directed to [insert details here]

**Signature of participant**	**Please PRINT name**	**Date**
____________________	____________________	______________
**Signature of witness**	**Please PRINT name**	**Date**
____________________	____________________	______________
**Signature of Research Coordinator**	**Please PRINT name**	**Date**
____________________	____________________	______________
